# Acremonium sp. diglycosidase-aid chemical diversification: valorization of industry by-products

**DOI:** 10.1007/s00253-023-12957-8

**Published:** 2024-03-02

**Authors:** Micaela Baglioni, Alexander Fries, Jan-Mathis Müller, Alejandra Omarini, Michael Müller, Javier D. Breccia, Laura S. Mazzaferro

**Affiliations:** 1 INCITAP-CONICET, FCEyN-Universidad Nacional de La Pampa (UNLPam), Av. Uruguay, 151 Santa Rosa, La Pampa Argentina; 2 Institut für Pharmazeutische Wissenschaften, Albert-Ludwigs-Universität Freiburg, Albertstraße 25, 79104 Freiburg, Germany; 3 Laboratorio de Biotecnología Fúngica y de los Alimentos. Asociación para el Desarrollo de Villa Elisa y Zona (ADVEZ), Héctor de Elia 1247, E3265 Villa Elisa, Entre Ríos Argentina

**Keywords:** Polyalcohols, Transglycosylation, Substrate specificity, Glycosylation/hydrolysis selectivity

## Abstract

**Abstract:**

The fungal diglycosidase α-rhamnosyl-β-glucosidase I (αRβG I) from *Acremonium* sp. DSM 24697 catalyzes the glycosylation of various OH-acceptors using the citrus flavanone hesperidin. We successfully applied a one-pot biocatalysis process to synthesize 4-methylumbellipheryl rutinoside (4-MUR) and glyceryl rutinoside using a citrus peel residue as sugar donor. This residue, which contained 3.5 % [w/w] hesperidin, is the remaining of citrus processing after producing orange juice, essential oil, and peel-juice. The low-cost compound glycerol was utilized in the synthesis of glyceryl rutinoside. We implemented a simple method for the obtention of glyceryl rutinoside with 99 % yield, and its purification involving activated charcoal, which also facilitated the recovery of the by-product hesperetin through liquid-liquid extraction. This process presents a promising alternative for biorefinery operations, highlighting the valuable role of αRβG I in valorizing glycerol and agricultural by-products.

**Keypoints:**

• *αRβG I catalyzed the synthesis of rutinosides using a suspension of OPW as sugar donor.*

• *The glycosylation of aliphatic polyalcohols by the αRβG I resulted in products bearing a single rutinose moiety.*

• *αRβG I catalyzed the synthesis of glyceryl rutinoside with high glycosylation/hydrolysis selectivity (99 % yield).*

**Graphical Abstract:**

**Figure:**
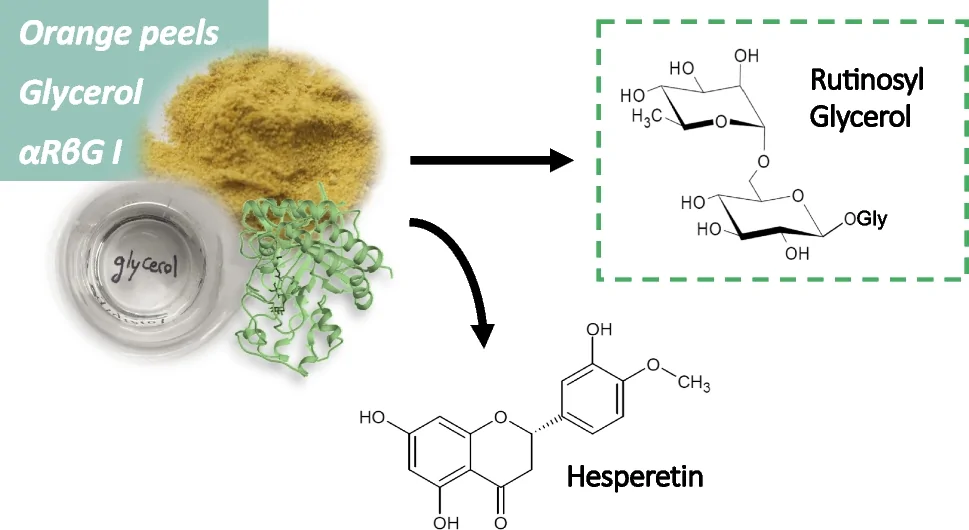

## Introduction

Food loss and waste (FLW) is a pervasive problem that occurs at all stages of the food system, from initial production, processing or derivatization, distribution, and consumption. The growing worldwide attention on FLW stems from its magnitude, given that approximately one-third of the food produced for human consumption is waste (Gustavsson et al. 2011; Dou and Toth 2021). This incredible imbalance between production and consumption represents one of the main factors contributing to the global environmental burden. In recent years, researchers have focused on the development of different processing methods for the full exploitation of various agricultural by-products and waste products.

Citrus is one of the most important crops around the world, not only because the fruit itself is consumed, but also because it is used to produce other products, such as fresh juices, citrus-based drinks, and jelly. Citrus peel wastes (CPW) remaining after processing oscillates between 50 and 70 % [w/w] of the processed fruits and considering the volumes handled, probably close to 10 million tons/year (Matsuo et al. 2022; Zema et al. 2018). Traditional CPW disposal strategies (e.g., incineration or landfilling) require high costs, and unauthorized disposal generates environmental problems since greenhouse gas (GHG) emissions and leachates are produced that can cause damage to the soil (due to the toxicity of essential oils on the soil microflora) and water body pollution (Ortiz-Sanchez et al. 2023; Zema et al. 2018).

Hesperidin is a 7-*O*-rutinosylated flavanone found in abundance in residues of citrus such as orange (Nayak et al. 2015; De la Rosa et al. 2018) and serves as sugar donor in the synthesis of glycoconjugates catalyzed by the diglycosidase α-rhamnosyl-β-glucosidase I (αRβG I) produced by the fungal strain *Acremonium* sp. DSM 24697. Since the enzyme shows substrate specificity for 7-*O*-rutinosylated flavanones (Mazzaferro and Breccia 2012), in this work, we aimed at the synthesis of rutinosylated compounds using citrus waste “as it is.” Regarding the sugar acceptor, the enzyme is rather unspecific, as primary or secondary, aliphatic or aromatic alcohols, are glycosylated (Weiz et al. 2019; Mazzaferro et al. 2010, 2012). Taking advantage of these enzyme properties, and with the focus on the valorization of products from different sources, we aimed at the synthesis of glyceryl rutinoside. Nowadays, the supply of glycerol is entirely independent of its demand, as there is as much glycerol as the amount of vegetable oils and animal fats are hydrolyzed to make oleochemicals or transesterified to produce biodiesel (Ciriminna et al. 2014). Too much surplus of crude glycerol impacted the refined glycerol market lowering the prices; thus, it is of great importance to find new applications for both refined and crude glycerol (Yang et al. 2012). Glycerol is a key cosmetic ingredient, as it is compatible with human skin having moisturizing and protective effects (Jungermann and Sonntag 1991; Mast 2018). Moreover, some natural glycosylated derivatives of glycerol have been proven to be potent osmolytes, an important characteristic for cosmetics industry, and a low-calorie sweetener, useful for food industry (Goedl et al. 2008). Even more, glycosides of glycerol could be used as a building block in the synthesis of glycoglycerolipids. These compounds showed a wide and hopeful spectrum of biological and pharmacological activities including anti-tumor, anti-viral, and anti-inflammatory activities (Zhang et al. 2014; Abedin and Barua 2021; Guo and Wang 2022).

In this work, we enzymatically synthesized glyceryl rutinoside from OPW and glycerol, and the resulting compound was purified. Moreover, we demonstrated that is also possible to synthesize the aromatic compound 4-methylumbellipheryl rutinoside (4-MUR) from OPW. These results indicate the potential of industrial wastes by making use of enzymes in the production of complex carbohydrate-based molecules.

## Materials and methods

### Chemicals and enzyme sources

All chemicals were from standard sources. Stock solutions of hesperidin (45 mM) were prepared using dimethyl formamide as the solvent. The enzyme αRβG I (EC 3.2.1.168) from *Acremonium* sp. DSM 24697 was recombinantly produced in *Pichia pastoris* and the supernatant used as enzyme source (Weiz et al. 2019).

### Characterization of orange peel waste (OPW)

OPW was provided by ECA Agroindustria (Concordia, Entre Ríos). The samples were dried to a constant weight through lyophilization. Klason lignin and cellulose were quantified following the method outlined by Bettucci et al. (1998). Hemicellulose content was determined gravimetrically using the procedure described by Breccia et al. (1995). Total sugar content in the biomass was analyzed using the micro-method reported by Masuko et al. (2005), while soluble sugars were measured using the method developed by Yemm and Willis (1954). Protein content was quantified using the Kjeldahl method for organic nitrogen (Bradstreet 1954). Acidic and neutral detergent fibers were determined based on the methodology described by Van Soest et al. (1991), and ethereal extract was quantified according to Saa-Otero et al. (2000). The quantification of hesperidin was carried out according to Mazzaferro and Breccia (2012). All assays were performed in triplicate, and the results were expressed as a percentage of the dry weight.

### Enzymatic hydrolysis activity

For quantification of αRβG I activity, each reaction contained 1.8 mM hesperidin, 4 % of DMF in 190 μL of 50 mM sodium citrate buffer pH 5.0, and 10 μL of suitably diluted enzyme solution. The reaction was performed for 10 min at 60 °C. One unit of αRβG I activity was defined as the amount of enzyme required to release 1 μmol of reducing sugars (as maltose) per min.

### Synthesis of 4-methylumbellipheryl rutinoside (4-MUR) using OPW

To perform the transglycosylation reaction using OPW, 0.3 g of lyophilized OPW was combined with 10 mL of a 1.8 mM solution of 4-methylumbelliferone in 50 mM sodium citrate buffer pH 5, with 0.03 U/mL of the enzyme αRβG I (30 °C, 20 min).

To analyze the resulting transglycosylation products, LC-MS was employed. The samples were subjected to centrifugation to separate the supernatant from the orange peels. Afterwards, 300 μl of methanol were added to 100 μL of the supernatant, mixed for 1 min, and centrifuged (4 °C, 20 min, 4 000 *g*). The analysis was conducted using an LC series Summit System (Dionex) coupled with an API2000 mass spectrometer (AB Sciex), employing an Isera ISAspher 100-3 C18-AQ-PoiE column (150 × 2 mm, 3 μm). For the mobile phase, a linear gradient of acetonitrile was utilized (flow rate: 0.25 mL/min, column temperature: 45 °C, injection volume: 10 μL); gradient program: ACN: 10 %, 0–2 min; 10 % → 90 %, 2–10 min; 90 % → 90 %, 10–10.5 min, 90 % → 10 %, 10.5–11 min; 10 % → 10 %, 11–19 min. The compounds were detected by UV-VIS and mass spectrometry. The following parameters for mass detection in positive ion mode were used (Q1 MS): ion source gas 1, 25 psi; ion source gas 2, 40 psi; curtain gas, 15 psi; ion spray voltage, 5 000 V; temperature, 400 °C; entrance potential, −10 V, declustering and focusing potential were used in screening mode.

### Transglycosylation of ethylene glycol and glycerol

For transglycosylation reactions, ethylene glycol and glycerol (40 % [v/v]) were incubated together with 1.8 mM hesperidin and 0.02 U/mL of αRβG I in 50 mM sodium citrate buffer pH 5 for 30 min at 60 °C. The transglycosylation reaction to glycerol and ethylene glycol were analyzed by LC-MS. The samples were prepared using Vivaspin 500 concentrators (Sartorius). The concentrate was then diluted (1:3) using 10 mM ammonium formate solution pH. At the end, acetonitrile was added up to a final concentration of 70 % [v/v]. The analysis was performed using an LC series 1100 System (Agilent) coupled to a triple quadrupole mass detector (QTrap 4500 mass spectrometer; AB Sciex) with an APS-HP column (3 μm, HILIC, 60 × 2 mm, Multospher). As mobile phase, an isocratic flow of 85 % acetonitrile and 15 % water was applied (flow rate: 0.3 mL/min, column temperature: 30 °C, injection volume: 2.5 μL). The following parameters for mass detection in negative ion mode were used (Q1 MS): ion source gas 1, 40 psi; ion source gas 2, 50 psi; curtain gas, 30 psi; ion spray voltage, −4 000 V; temperature, 400 °C; declustering potential, −70 V; focusing potential, −260 V; entrance potential, −10 V.

### Glyceryl rutinoside synthesis optimization

To quantify the concentration of the transglycosylation product(s), rutinose (*n*_Rutinose_) and hesperetin (*n*_Hesperetin_) were quantified (Miller 1959, Mazzaferro and Breccia 2012) using an Epoch Microplate Spectrophotometer (BioTek) together with 96-well UV Flat Bottom Microtiter Plates (Thermo).

Schematically, the enzymatic reactions catalyzed by αRβG I are the following:

1. Hesperidin + H_2_O ➔ Hesperetin + Rutinose (hydrolysis)
2. Hesperidin + R-OH ➔ Hesperetin + R-rutinoside (transglycosylation)

When the reaction is conducted without alcohol, only reaction (1) takes place and the amounts of products (*n*, mol) correlate as follows:

$${n}_{Hesperetin}={n}_{Rutinose}$$

$${n}_{R- rutinoside}=0$$

However, when the reaction is conducted with added alcohol, and because water cannot be excluded from the reaction medium, both reactions (1) and (2) take place, and the amounts of products correlate as follows:

$${n}_{Hesperetin}={n}_{Rutinose}+{n}_{R- rutinoside}$$

Therefore, the difference between the hesperetin and the rutinose concentrations was taken as a measure for the amount of transglycosylation product in the measured sample.

To optimize the synthesis reaction, several parameters were evaluated. Initially, the glycerol concentration was investigated over a range of 0 % to 70 % [v/v]. The reaction mixture comprised 1.8 mM hesperidin in a 50 mM sodium citrate buffer pH 5, with 6 % [v/v] DMF and 0.03 U/mL of αRβG I (60 °C, 30 min). Subsequently, the reaction temperature was assessed in the range of 15 to 80 °C, utilizing a reaction mixture containing 50 % [v/v] glycerol. Following that, the enzyme quantity was examined within the range of 0.02 to 0.2 U/mL, using a reaction mixture containing 50 % [v/v] glycerol. The temperature was maintained at 60 °C, and the reaction time was reduced to 15 min to observe any variations. Finally, the reaction progress was monitored over time, using the optimized conditions. All experiments were performed in duplicate.

### Transglycosylation of glycerol from OPW and purification

To perform the transglycosylation reaction using OPW, 0.9 g of OPW lyophilizate was mixed with 30 mL of 50 % [v/v] glycerol in 50 mM buffer sodium citrate pH 5, and 0.09 U/mL of αRβG I was used for the reaction (60 °C, 17 h). Then, the enzyme was inactivated placing the sample boiling-water bath (10 min) and centrifugated (10 min) to separate the liquid phase.

A solvent extraction was performed three times by adding 0.5 volume of ethyl acetate. The product solution (15 mL aqueous phase) was diluted with 35 mL demineralized water. The solution was then incubated together with 1.5 g of activated charcoal under constant stirring (room temperature, 30 min) and filtered (filter paper Double Rings 103).

The activated charcoal was washed four times with 50 mL demineralized water (constant stirring, room temperature, 5 min) and filtrated. To elute the glyceryl rutinoside from the surface of the charcoal, 50 % [v/v] ethanol was added (constant stirring, room temperature, 30 min) and filtered. The process was performed three-times. The ethanol was evaporated (60 °C), and the remaining solution was analyzed by direct infusion mass spectrometry. The mass spectrometry experiments were performed in a XEVO-G2XSQTOF system using the electrospray (ESI) source. The samples were introduced into the mass spectrometer at 10 μL/min flow rate using a direct infusion mode. Data were obtained in positive ionization mode, acquiring full scan spectra for 1 min in the *m/z* range 50–1 000 with 1 s scan time. ESI-QToF positive mode parameters: capillary, 3 kV; sampling cone 38 V; source temperature 100 °C; source offset 25 V; desolvation temperature, 250 °C; cone gas flow, 50 L/h; desolvation gas flow 600 L/h. To acquire the MS/MS spectra from the ion at *m/z* 423, nitrogen was used as collision gas with a collision energy of 37.8 eV.

## Results

### Characterization of orange peel waste (OPW)

When citrus fruits are processed for juice or essential oil extraction, nearly 50 % of fruit wet mass is comprised of peel residues (Negro et al. 2016). In this work, orange peel waste was characterized in terms of the structural components cellulose, lignin and hemicellulose, and soluble sugars (Table 1). Regarding the polyphenolic compounds, the total polyphenol content (2.9 mg gallic acid equivalents/g dry weight, ca. 1 % [w/w] converted to hesperidin) was apparently lower than the concentration of hesperidin (3.5 ± 0.5 % [w/w dry weight]), the mayor flavonoid present in OPW (Ortiz-Sanchez et al. 2023). However, it is worth noting that hesperidin quantification was carried out through heterogeneous catalysis with the enzyme αRβG I from *Acremonium* sp. DSM 24697, while the quantification of polyphenols was performed on the supernatant of the OPW water suspension. These differences may be attributed to the fact that enzymatic catalysis favors the extraction/separation of hesperidin from the solid matrix. This was confirmed by quantifying the total polyphenols in an aqueous extract with and without enzymatic treatment (αRβG I and II and combining both) in which the total polyphenol content increased in all treatments (data not shown). Therefore, the transglycosylation assays using OPW as sugar donor was conducted in the presence of the suspension rather than solely using the supernatant.

**Table 1:** Chemical characterization of OPW (% w/w, dry weight)

Feature	Concentration
Cellulose	20.45 ± 0.45
Hemicellulose	26.00 ± 2.82
Klason lignin	7.09 ± 0.37
Acid soluble lignin	1.47 ± 0.25
Total sugars	1.28 ± 0.09
Soluble sugars	0.013 ± 0.001
Crude protein	8.11 ± 0.13
Acid detergent fiber	29.07 ± 0.63
Neutral detergent fiber	38.11 ± 0.34
Etheric extract	12.02 ± 0.23
Hesperidin	3.5 ± 0.56

### Synthesis of 4-methylumbelliferyl rutinoside using OPW

**Scheme 1:**
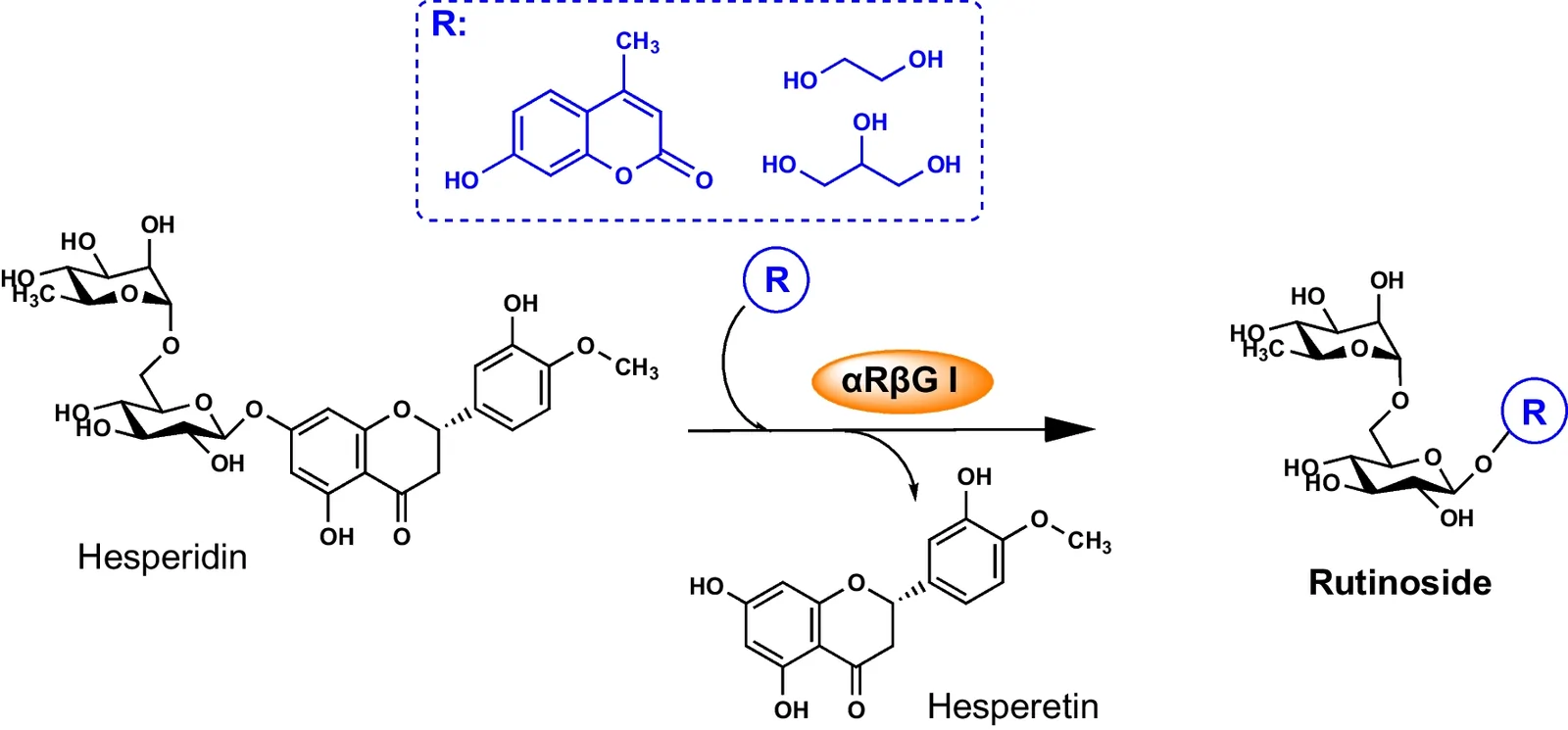
Transglycosylation reaction from hesperidin to various OH-acceptors performed with the diglycosidase αRβG I from *Acremonium* sp. DSM 24697

**Fig. 1:**
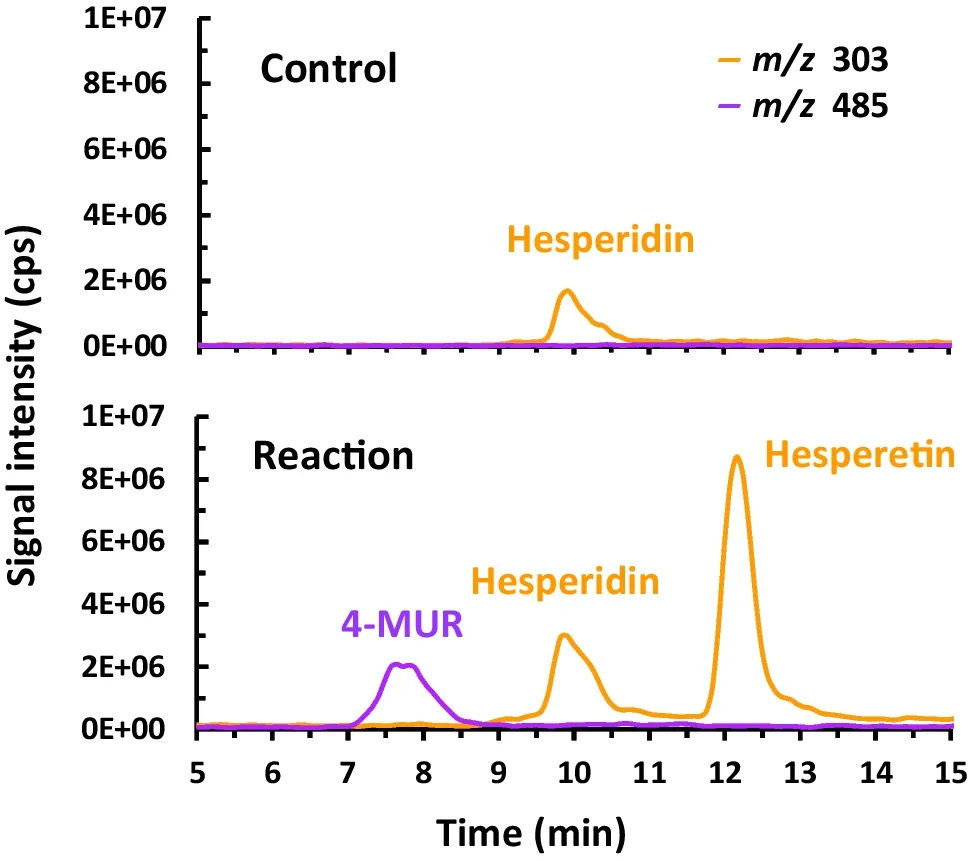
LC-MS analysis of the 4-MUR synthesis reaction using OPW as hesperidin source, 4-MU as sugar acceptor and the enzyme αRβG I as catalyst. The control was performed in the same conditions with inactivated enzyme. The products were detected by single-ion monitoring in positive ion mode at *m/z* 303 (orange curve) and 485 (violet curve) for the [M + H]^+^ ions of the products hesperetin (Rt 12.3 min) and 4-MUR (R_t_ 7.8 min), respectively

The capability of OPW to act as a sugar donor in the synthesis of aromatic glycoconjugates was evaluated by transglycosylation with αRβG I of 4-methylumbelliferone (4-MU) as sugar acceptor (Scheme 1), and the reactions were analyzed by HPLC-MS. The synthesis product 4-MUR is a fluorogenic substrate for screening of rutinosidase activity by zymographic staining and has been recently demonstrated to possess therapeutic effect against hepatocellular carcinoma models (Mazzaferro et al. 2012; Weiz et al. 2022). The mass spectrometric analysis revealed signals corresponding to the [M + H]^+^ ions of the reaction products hesperetin (*m/z* 303, R_t_ 12.3 min) and the desired compound 4-MUR (*m/z* 485, R_t_ 7.8 min) (Fig. 1). A signal at *m/z* 303 was also observed for the substrate hesperidin (R_t_ 9.8 min) corresponding to the ion source fragmentation of the molecule at the heterosidic linkage. That means, the enzyme αRβG I was able to catalyze the synthesis of the glycoconjugate 4-MUR using raw materials in suspension.

### Mass spectrometry analysis of polyalcohols transglycosylation products

**Fig. 2:**
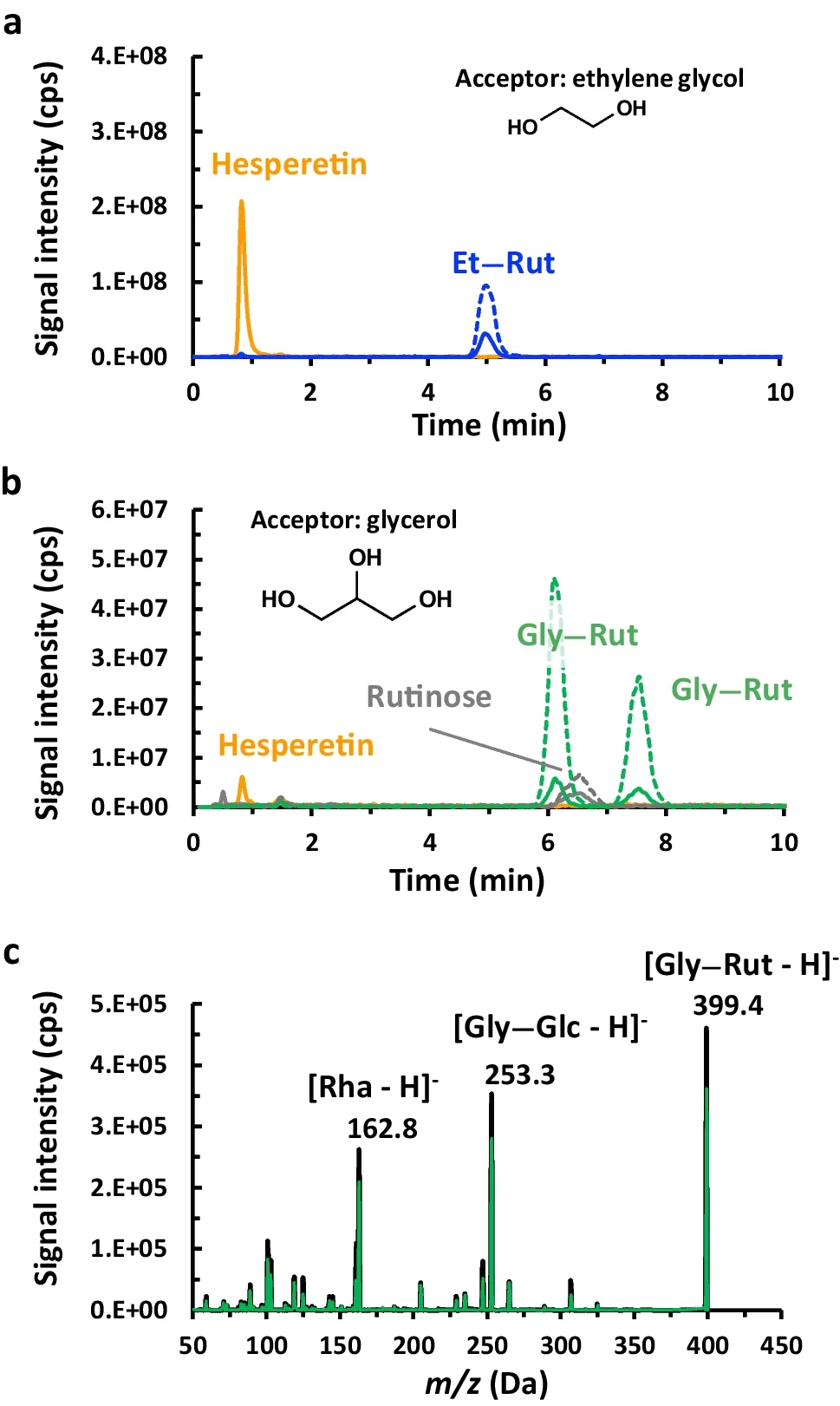
LC-MS analysis of the reactions catalyzed by αRβG I with 40 % [v/v] **a** ethylene glycol and **b** glycerol as sugar acceptors. The ions were detected at *m/z* 301 for hesperetin (orange curve), 325 and 371 for rutinose (gray curve), 369 and 415 for ethylene glycol rutinoside (blue curve), and 399 and 445 for glyceryl rutinoside (green curve). The solid lines correspond to the [M - H]^-^ ion and the dashed lines to the [M - H + HCO_2_H]^-^ adduct. **c** MS/MS spectra of the [M - H]^-^ ions with *m/z* 399 with R_t_ 6.2 min (black line) and 7.6 min (green line) of the sample using glycerol as sugar acceptor

To evaluate the transglycosylation of polyalcohols, glycerol and ethylene glycol were tried as sugar acceptors, and pure hesperidin was used as the substrate (Scheme 1). The formation of products was detected by liquid chromatography with mass spectrometry detection (LC-MS). For ethylene glycol as the sugar acceptor, at least two products with the *m/z* values corresponding to hesperetin (R_t_ 0.82 min) and ethylene glycol rutinoside (R_t_ 5.0 min) were observed (Fig. 2a, Table 2). Only traces of rutinose could be detected. No peaks were found with the *m/z* value corresponding to ethylene glycol rutinoside. For glycerol as sugar acceptor, signals could be observed with masses corresponding to the ion substrate hesperidin (*m/z* 609, R_t_ 1.46 min), its aglycone hesperetin (*m/z* 301, R_t_ 0.82 min) and rutinose (*m/z* 325 and 371, R_t_ 6.53 min). For the mass corresponding to glyceryl rutinoside (*m/z* 399 and 445*)*, two peaks could be observed (R_t_ 6.2 min; 7.6 min) (Fig. 2b, Table 2). No signals were observed corresponding to the mass of glyceryl dirutinoside or glyceryl trirutinoside. Therefore, under the given conditions, the αRβG I was capable of transglycosylating polyalcohols only once.

**Table 2:** Expected masses and observed *m/z* and retention times for the substrates and products of the transglycosylation reaction to polyalcohols. *ND* not detected

Compound	Exact mass (Da)	[M - H]^-^	[M - H + HCO_2_H]^-^	R_t_ [min]
Hesperidin	610.19	609.20	---	1.46
Hesperetin	302.08	300.50	---	0.82
Rutinose	326.12	325.10 (traces)	371.20 (traces)	6.53
Ethylene glycol	62.04	---	---	---
Ethylene glycol rutinoside	370.15	369.10	415.0	5.0
Ethylene glycol dirutinoside	678.26	ND	ND	---
Glycerol	92.05	---	---	---
Glyceryl rutinoside	400.16	399.30	445.10	6.2; 7.6
Glyceryl dirutinoside	708.27	ND	ND	---
Glyceryl trirutinoside	1016.38	ND	ND	---

To differentiate the two peaks corresponding to the mass of glyceryl rutinoside, the MS/MS spectra were obtained (Fig. 2c). The spectra show, however, the same fragmentation pattern. The different retention times observed for these peaks, combined with their identical fragmentation pattern, suggest that these two peaks correspond to regioisomers, i.e., the primary or the secondary hydroxy groups could act as sugar acceptors. In sum, the glycosylation of aliphatic polyalcohols by the αRβG I resulted in products bearing a single glycosidic moiety.

### Optimization of the synthesis of glyceryl rutinoside

In order to obtain the reaction conditions under which the transglycosylation reaction has its highest product outcome, different conditions were evaluated.

**Fig. 3:**
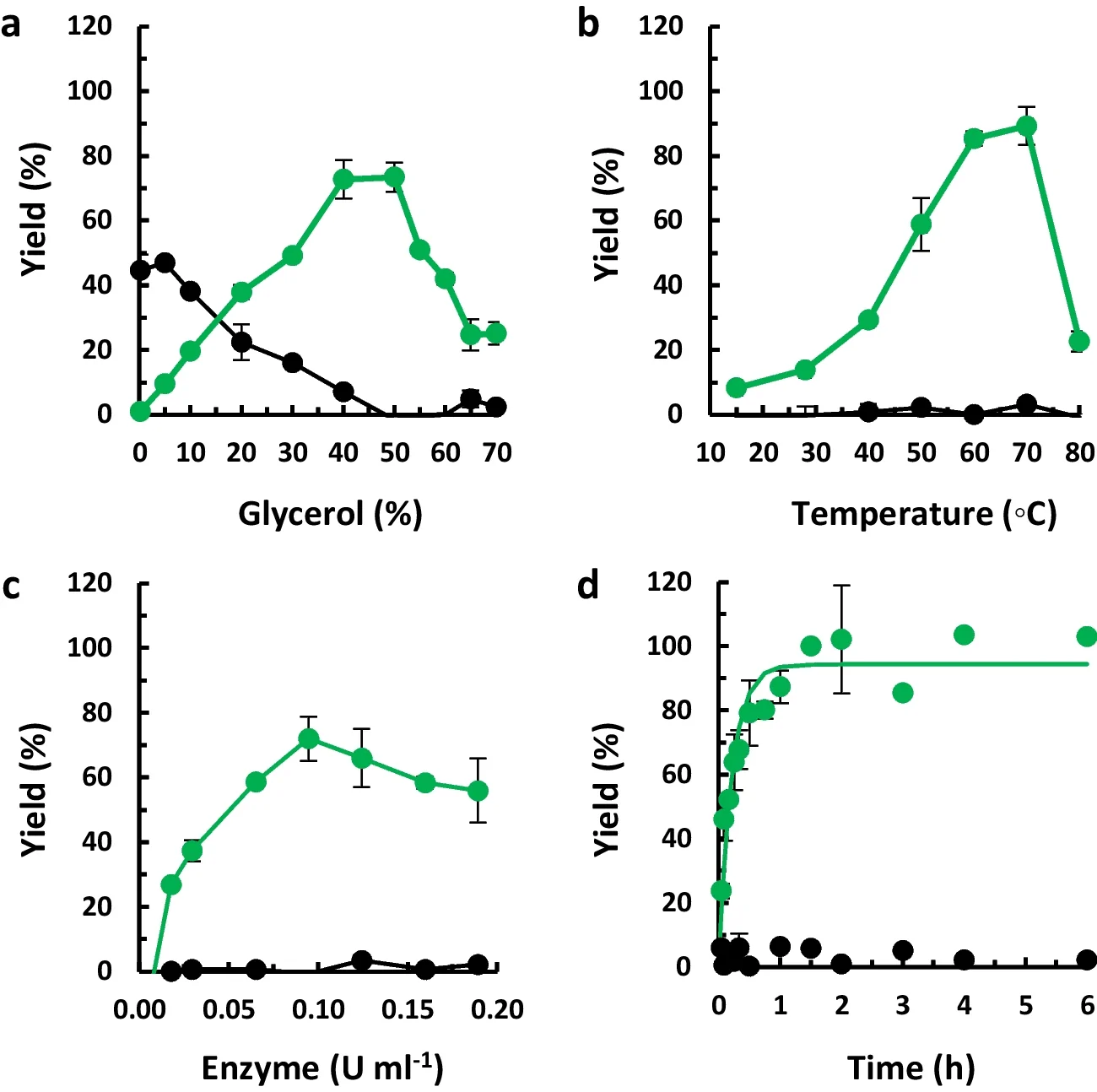
Effect of **a** glycerol concentration, **b** temperature, **c** enzyme concentration, and **d** time course of the glyceryl rutinoside synthesis catalyzed by αRβG I. Quantification of hydrolysis (black) and transglycosylation yield (green)

The effect of glycerol concentration was evaluated within the concentration range of 0–70 % [v/v] (Fig. 3a). Since the reactions are performed in aqueous medium, there is competition between water and glycerol as sugar acceptors, leading to rutinose and glyceryl rutinoside, respectively. In both cases, hesperetin is also a reaction product. When no glycerol was added, a yield of 45 % rutinose was observed. With increasing glycerol concentration, the conversion to rutinose continuously decreased, with no detectable hydrolysis at concentrations equal to or greater than 50 % [v/v] glycerol.

The yield of glyceryl rutinoside reached a maximum between 40 and 50 [v/v] glycerol. For concentrations greater than 55 % [v/v] glycerol, the yield decreased, possibly related to the higher viscosity. The concentration of 50 % [v/v] glycerol was chosen for further experiments, as it resulted in maximum transglycosylation yield (73 %), while the sugar rutinose was not detected (<1 %). In other words, the reaction was selective towards the transglycosylation in such conditions.

The conversion of hesperidin to glyceryl rutinoside improved with increasing temperatures with maximum yield (ca. 85 %) at 60–70 °C, while the rutinose concentration remained under 1 % (Fig. 3b). Considering the energetic differences required for the process, a temperature of 60 °C was selected for future assays. Regarding the enzyme concentration (0.02–0.2 U/mL), the reaction time was lowered to half (15 min) in order to be able to evaluate differences. The maximum yield was 72 % using 0.09 U/mL enzyme (Fig. 3c). Furthermore, the time course of the reaction was monitored under the previously optimized reaction conditions (50 % [v/v] glycerol, 60 °C, and 0.09 U/mL). The concentration of glyceryl rutinoside increased over time and reached more than 99 % yield after 90 min. Finally, considering that transglycosylation reactions can generate products susceptible to subsequent hydrolysis by the same enzyme (Hansson et al. 2001; Mangas-Sánchez and Adlercreutz 2015), the products concentration continued to be monitored. After 6 h of reaction, the product concentrations remained unchanged (Fig. 3d), suggesting that they do not act as substrates for the enzyme under the tested conditions.

Biocatalytic process and purification of glyceryl rutinoside with OPW as sugar donor

**Fig. 4:**
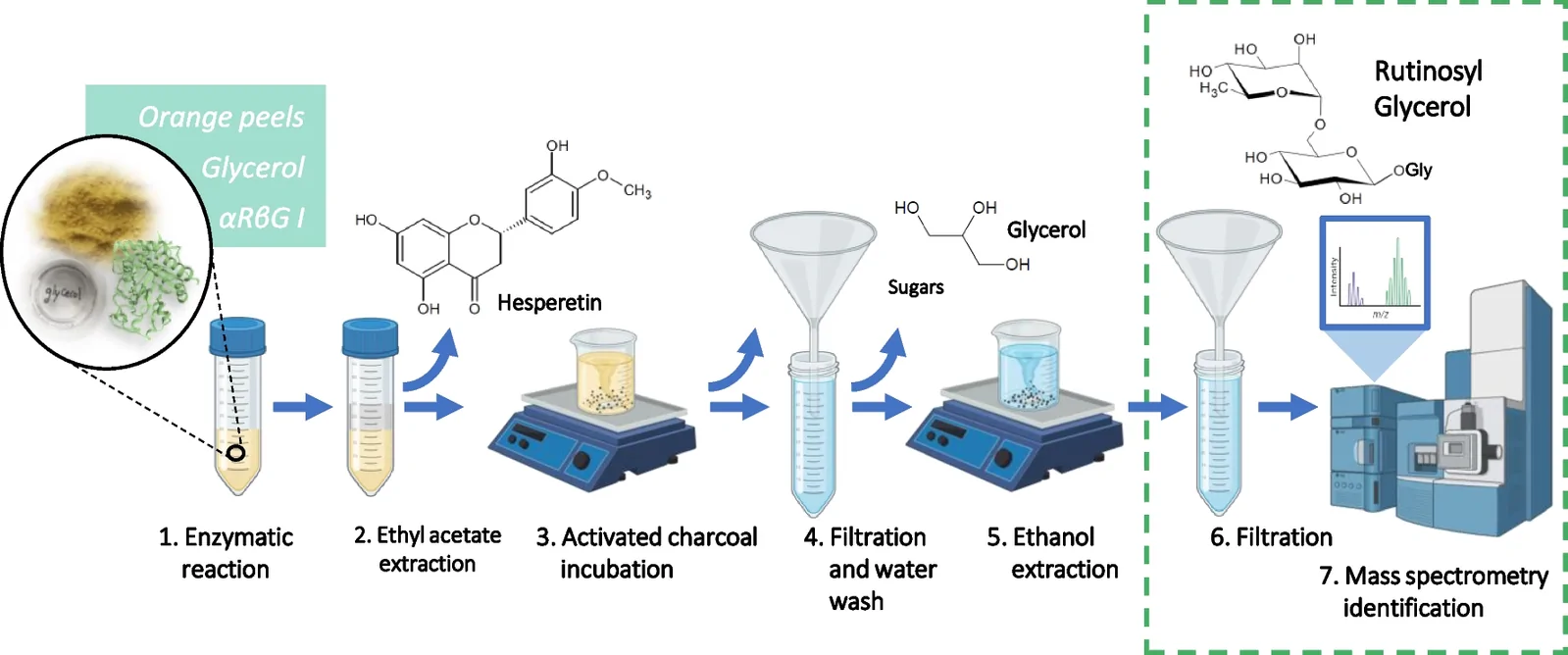
Synthesis of glyceryl-rutinoside from glycerol and OPW using αRβG I: biocatalytic process, hesperetin recovery and purification

**Fig. 5:**
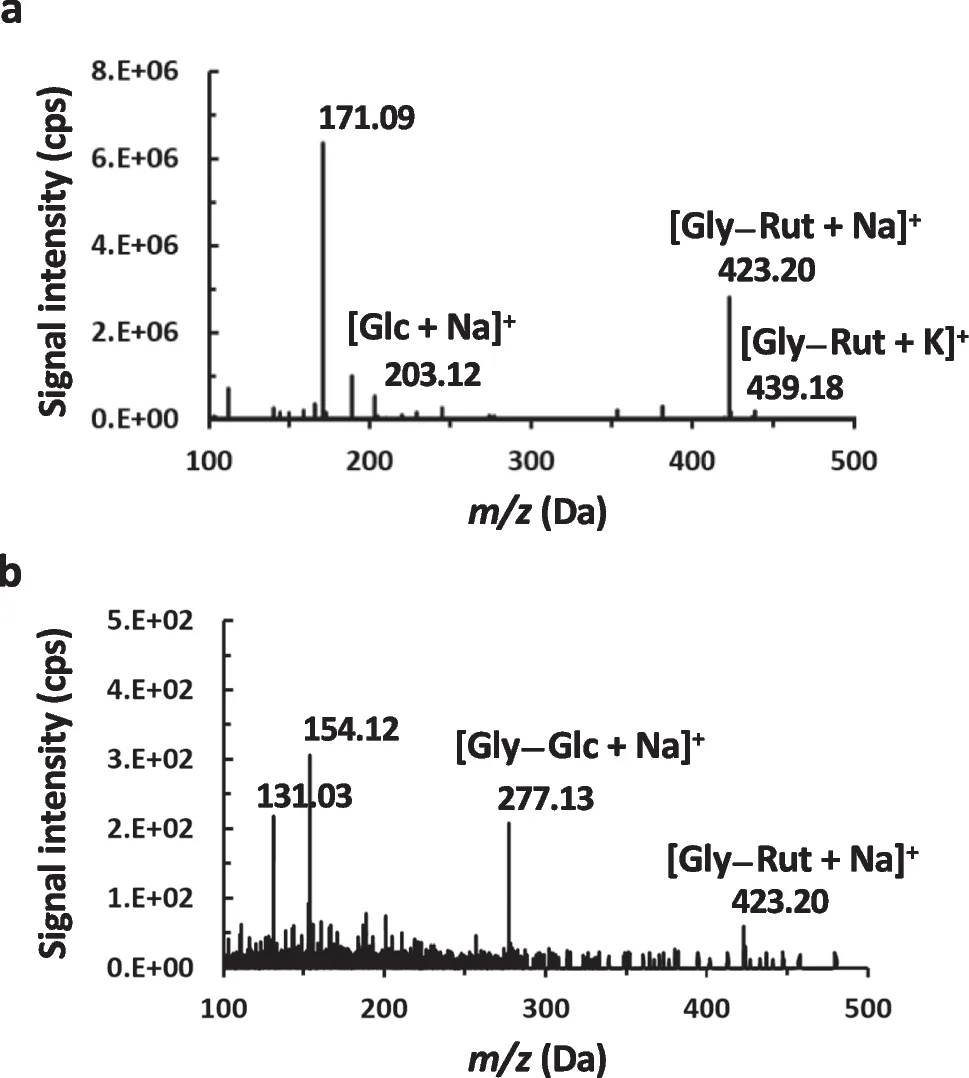
Mass spectrometry analysis of purified glyceryl rutinoside by direct infusion (ESI-QToF)

Enzymatic synthesis of glyceryl rutinoside was carried out under optimized reaction conditions (50 % [v/v] glycerol, 60 °C, 0.09 U/mL of the enzyme αRβG I), using a suspension of dried OPW (0.3 g/mL) as the hesperidin source (Fig. 4). To purify the transglycosylation product, the solids were removed by centrifugation after the reaction. Subsequently, a liquid-liquid extraction was conducted using ethyl acetate to separate and recover the reaction product hesperetin. This step facilitated its retrieval as a high-value product in a separate side-stream. Due to their hydrophilic character, free sugars from OPW, glycerol, and the rutinosylated products remained in the water phase. This solution was incubated with 3 % [w/v] activated charcoal and washed with water. The free sugars as well as glycerol remained in the water phase, as evidenced by quantification of reducing sugars and by thin-layer chromatography analysis (data not shown). Finally, glyceryl rutinoside was eluted from activated charcoal with 50 % [v/v] ethanol. It is important to mention that the selective binding of glyceryl rutinoside was accomplished with 3 % [w/v] activated charcoal; using 45 % [w/v] charcoal caused the binding of the carbohydrates present in the sample, preventing the correct purification of glyceryl rutinoside (data not shown). The purified glyceryl rutinoside was analyzed by direct infusion mass spectrometry (QToF detector). In the positive ion mode, the mass spectrum displayed ions corresponding to the sodium [M + Na]^+^ and potassium [M + K]^+^ adducts at *m/z* 423 and 439, respectively (Fig. 5a). For further MS/MS analysis, the sodium adduct was chosen. A predominant fragmentation pattern associated with the cleavage of the glycosidic bond between glucose and rhamnose (Fig. 5b) was supported by the ion at *m/z* 277, which is likely derived from the elimination of a rhamnose residue. The ion at *m/z* 131 might be a fragment originating from a glucose residue. In summary, the biocatalytic process and purification enabled the production of the synthesis product glyceryl rutinoside, alongside with hesperetin.

## Discussion

The enzyme αRβG I was able to catalyze the synthesis of the glycoconjugates 4-MUR and glyceryl rutinoside using the raw material OPW in suspension, instead of pure hesperidin. This achievement was made possible due to the specificity of the enzyme towards the sugar donor hesperidin, acting in a complex mixture of polysaccharides. Comparatively, the enzymatic preparation derived from *Penicillium multicolor* Aromase H2, the only commercially available diglycosidase, was successfully utilized for synthesizing diglycosylate tyrosol (Haluz et al. 2023). This synthesis was possible because the mixture comprises a specific diglycosidase activity, β-acuminosidase, while having undetectable levels of β-apiosidase. In principle, this preparation could be applied to the synthesis of rutinosylated compounds, as hesperidin and rutin can act as substrates. However, the high levels of rhamnosidase activity (twofold in comparison with β-rutinosidase activity, using pNP-derivatives as the substrates) (Haluz et al. 2023), precludes its application, as it could reduce the concentration of available hesperidin or hydrolyze glyceryl rutinoside at the rhamnosidic linkage. This enzymatic preparation contains also several monoglycosidases (α-glucosidase, β-glucosidase, α-galactosidase, β-galactosidase, α-arabinofuranosidase, α-arabinopyranosidase, α-xylosidase) and diglycosidases (β-acuminosidase, β-vicianosidase and β-primeverosidase) (Wan et al. 2019; Haluz et al. 2023), which might lead to secondary reactions yielding undesired products or degradation of the product of interest. Additionally, OPW poses a challenge due to its abundant soluble sugar content (Table 1), which can inhibit glycosidases (Brás et al. 2014). Nevertheless, αRβG I exhibited successful catalysis of the syntheses under these conditions, showcasing its potential for overcoming inhibitory effects and efficiently producing the desired glycoconjugates. In contrast to the specificity of the enzyme towards the sugar donor, its relaxed sugar acceptor specificity allowed it to accept compounds like 4-MU, ethylene glycol, and glycerol, leading to the formation of the corresponding glycoconjugates. Notably, when aliphatic polyalcohols were used, the glycosylation by the αRβG I resulted in products bearing a single glycosidic moiety, indicating a selective transglycosylation in such conditions. The presence of two peaks for the *m/z* corresponding to glyceryl rutinoside (Fig. 2b) suggested the glycosylation at different positions. Analogous results have been found with the synthesis of α-glyceroglucosides (α-GGs) carried out using the commercial α-glucosidase “transglucosidase L-AMANO” from *Aspergillus niger*. The reaction involved maltose and glycerol as substrates, resulting in a maximum yield of 66 % in the form of a mixture of three isomers: (2*R*)-1-*O*-α-GG, (2*S*)-1-*O*-α-GG, and 2-*O*-α-GG, with a ratio of isomers as 49:41:10 (Takenaka and Uchiyama 2000). Likewise, the enzymatic synthesis of β-glyceroglucosides (β-GGs) using cellobiose and glycerol as substrates and employing a β-glucosidase from *Pyrococcus furiosus* resulted in a mixture comprising 1-*O*-β-GG (79 %) and 2-*O*-β-GG (21 %) (Schwarz et al. 2009).

Glycosylation/hydrolysis selectivity in a water-based system and product inhibition are key restrictive factors impacting glycosylation efficiency, as underlined by the mechanistic studies of the α-glucosidase Agl2 from *Xanthomonas campestris* (Chen et al. 2019). In the synthesis of glyceryl rutinoside by αRβG I, we directed the reaction towards the glycosylation by varying the ratio of glycerol/water, with no detectable hydrolysis at glycerol concentrations higher than 50 % [v/v] (Fig. 3a). With 50 % [v/v] glycerol, the molar concentration of water remains four times higher than that of glycerol, demonstrating the selectivity of the enzyme towards the synthesis reaction. The concentration of glyceryl rutinoside increased over time and reached more than 99 % yield in a 90-min reaction, which did not decrease with longer reaction times, suggesting that the product does not act as substrate for the enzyme under the tested conditions (Fig. 3d). This behavior aligns with previous research involving terpenoids as sugar acceptors, where nerol, geraniol, and 2-phenylethyl alcohol rutinosylations had high yields (58 %, 76 %, and 89 %, respectively, based on the amount of donor added), and hydrolysis was not observed in an aqueous medium (Minig et al. 2011). In contrast, when the phenolic compounds hydroquinone and 4-methylumbelliferone were used as sugar acceptors, lower yields were obtained (38 % and 16 % yield regarding the sugar donor, respectively) (Mazzaferro et al. 2012, 2019). This behavior has been attributed to the hydrolysis of the synthesized compounds, requiring a kinetic control of the enzymatic process to achieve optimal yields (Mazzaferro et al. 2012, 2019). The enzymatic glycosylation involving the α-glucosidase Agl2 from *Xanthomonas campestris* exhibited remarkable glycosylation/hydrolysis selectivity for phenol as the sugar acceptor, in comparison to vanillin and ethyl vanillin (Chen et al. 2019). Notably, during extended reaction times, 40 % degradation was observed for vanillin and ethyl vanillin glycosides, while the degradation of phenol glycoside was comparatively lower (16 % degradation) (Chen et al. 2019). In other words, the glycosylation/hydrolysis selectivity not only impacts the synthetic yield but also plays a crucial role in determining the ease of reaction control.

In sum, three products with high added value, namely 4-MUR, glyceryl rutinoside, and hesperetin, were obtained by biocatalysis utilizing OPW. The biocatalytic process leading to the obtention of glyceryl rutinoside (alongside hesperetin), in conjunction with the ease of purification, could be a feasible alternative for a biorefinery process. Our results demonstrate the general applicability of citrus waste as hesperidin source for αRβG I-catalyzed processes and provide a sustainable approach to valorize agricultural by-products.

## Data Availability

The authors declare that all data generated or analyzed during this study are included in this published article. Should any raw data files be needed in another format, they are available from the corresponding author upon reasonable request.
